# Cholecystectomy and duodenogastric reflux: interacting effects over the gastric mucosa

**DOI:** 10.1186/s40064-016-3641-z

**Published:** 2016-11-14

**Authors:** Erdinc Mercan, Ugur Duman, Deniz Tihan, Evren Dilektasli, Kazim Senol

**Affiliations:** 1 Department of General Surgery, Yuksek Ihtisas Training and Research Hospital, Bursa, Turkey; 2 Department of General Surgery, Bursa State Hospital, Bursa, Turkey

**Keywords:** Laparoscopic cholecystectomy, Reflux gastritis, *H. pylori*

## Abstract

**Aims:**

To evaluate association between duodenogastric reflux and early gastric mucosal changes before and after the cholecystectomy procedure.

**Materials and methods:**

Patients were evaluated with preoperative and postoperative endoscopy and endoscopic biopsy. Demographic and clinical characteristics, histological parameters, presence of duodenogastric reflux, and Updated Sydney scores were noted.

**Results:**

A total of fifty patients who obeyed the follow-up were enrolled into the study. Median age of the patients was 43 years (range 25–84). Male–female ratio was 0.51 (17/33). Duodenogastric reflux % and Updated Sydney scores before and after cholecystectomy were 24 (48%) versus 39 (78%) and 2.38 ± 2.21 versus 3.46 ± 3.05, respectively (p = 0.001, p < 0.000). Mucosal inflammation degree showed significant increase in 15 (30%) patients, decrease in 7 (14%) patients and equality in 28 (56%) patients (p = 0.037). Neutrophil activation degree was significantly higher in 21 (42%) patients, lower in 5 (10%) patients after the surgery (p = 0.005). Postoperative glandular atrophy degree was also higher in 13 (26%) patients and equal in 37 (74%) patients (p = 0.001). Pre- and postoperative degree of intestinal metaplasia and *H. pylori* density did not any show significant difference (p = 0.157, p = 0.248, respectively).There were significant positive correlation between postoperative *H. pylori* infection and mucosal activity, inflammation, atrophy and intestinal metaplasia.

**Conclusion:**

Cholecystectomy is a potent inducer of pathologic duodenogastric reflux. Early onset of duodenogastric reflux and underlying *H. pylori* gastritis cause early gastric mucosal injury following cholecystectomy procedure by interacting collectively.

## Background

Cholelitiasis is one of the most common problems affecting the digestive tract. Although the incidence depends on the factors including age, gender and ethnicity, 10–15% of the adult population has the gall bladder stones (Schafer et al. 1998). Therefore, cholecystectomy procedure is the second most practiced surgical procedure.

Majority of the symptoms resolve after the cholecystectomy procedure. However, 15–20% of the patients present with new gastrointestinal symptoms or have continuous preoperative complaints (Manifold et al. 2000). Following the cholecystectomy procedure, the loss of reservoir function of gall bladder causes impairment in cyclic pattern of bile juice excretion; the loss of neurohumoral responds also causes motility changes in upper gastrointestinal system and should lead to increased duodenogastric reflux (DGR) (Perdikis et al. 1994). Thus, regurgitation of duodenal contents into the stomach induces the gastric mucosal injury resulting in persistent symptoms such as epigastric pain, nausea and bilious vomiting (Buxbaum 1982; Brough et al. 1984).

Pathologic DGR is observed in 51–89% of the patients after the cholecystectomy procedure (Fall et al. 2007; Chen et al. 2010). During the postoperative period, it has been demonstrated that bile refluxate levels and DGR severity increase in significant correlation with progressive atrophic gastritis (Lorusso et al. 1992). To reveal the underlying causes of gastric mucosal changes regarding post-cholecystectomy DGR, patients are evaluated with endoscopic examination and histological sampling. Reflux Gastritis Score (RGS) (Dixon et al. 1986) and Biliary Reflux Index (BRI) (Dixon et al. 2001) scoring systems have been established to identify the degree of mucosal injury and reactive gastritis. However, RGS and BRI scoring systems are unable to demonstrate the atrophic gastric mucosal changes due to long-term exposure of bile refluxate. Updated Sydney scoring system has the superiority over these scoring systems by providing the glandular atrophy and intestinal metaplasia degree (Dixon et al. 1996). In the literature, there are few studies attempted to evaluate the post-cholecystectomy DGR and mucosal atrophy related to DGR with Updated Sydney scoring system (Lin et al. 2009; Kellosalo et al. 1991; Zullo et al. 1998).

In this prospective observational cohort study, we aimed to identify the DGR and early atrophic changes of the gastric mucosa before and after cholecystectomy procedure with Updated Sydney scoring system.

## Methods

This prospective cohort study was conducted in a referral gastrointestinal endoscopy unit of Bursa Yuksek Ihtisas Training and Research Hospital between January 2012 and March 2013. Institutional ethics committee approved the clinical research with 10,877 approval code. All patients provided written informed consent. Patients requiring cholecystectomy procedure due to symptomatic gallstone disease (episodic pain in epigastrium and right upper quadrant, bloating and belching associated with the pain vs.) were enrolled into the study. All of the patients were evaluated with Esophagogastroduodenoscopy (EGD) and endoscopic biopsy 30 days before the surgery. Postoperative EGD was performed to display the improvements of duodenogastric reflux (DGR) and histological alterations 60 days after the surgery. Observation of active bile reflux, presence of bilious gastric lake and gastritis were noted. Antral biopsies were taken 2–3 cm over the pylorus along the lesser and greater curvature. The specimens were placed in a 10% formalin solution, embedded in paraffin blocks and stained with hematoxylin/eosin Alcian-blue (AB, pH 2.5)/periodic acid Schiff (PAS). Histological parameters including chronic inflammation, neutrophil activation, glandular atrophy, intestinal metaplasia and presence of *H. pylori* were evaluated. Gastric mucosal changes and microbiological aspects of the chronic gastritis were reviewed with Updated Sydney classification system and graded into absent, mild, moderate and severe disease. Demographic data and clinical parameters of the patients were also recorded.

Patients with chronic co-morbid diseases (diabetes mellitus, coronary artery disease, and hypertension), long-term non-steroidal analgesic and oral contraceptive drug use, previous history of biliary and/or gastric surgery, gastric malignancy and ulcer, cholecystectomy during pregnancy, conversion to open surgery were excluded from the study.

Early morphologic changes of gastric mucosa and evidence of enterogastric reflux after laparoscopic cholecystectomy was determined as the main clinical outcome of this study.

### Statistical analyses

Shapiro–Wilk test were used to assess normality. The normally distributed data was presented as mean ± SD (standard deviation) and non-normally distributed data was presented as median value (interquartile range). Baseline characteristics were compared with paired Student’s *t* test or the Wilcoxon Signed-rank test for continuous variables or Mcnemar’s test for categorical variables. The Wilcoxon signed-rank test was also used to assess differences before and after the surgery in each group. The Spearman’s rank correlation was used to define a correlation between histological parameters and DGR. All statistical procedures were performed with SPSS 15.0 (SPSS Inc, Chicago, Illinois). p < 0.05 was considered as significant.

## Results

50 patients who evaluated with pre- and post-cholecystectomy endoscopy and attended follow-up regularly were enrolled into the study. Median age of the patients was 43 years (range 25–84). Male–female ratio was 0.51 (17/33). Cholecystectomy etiology was stones in all patients. Mean time of preoperative esophagogastroduodenoscopy (EGD) and endoscopic biopsy were 38 ± 8 days. Preoperative endoscopy revealed duodenogastric reflux (DGR) in 24 (48%) patients and *H. pylori* infection in 14 (28%) patients. Updated Sydney score mean value before surgery was 2.38 ± 2.21 (range 0–11). Demographic and clinical characteristics of patients were demonstrated in Table 1. Laparoscopic cholecystectomy was successfully performed in all patients without any complication. Postoperative follow-up was uneventful and all patients were discharged on postoperative first day. Mean time of postoperative EGD and endoscopic biopsy were 56 ± 10 days. Postoperative EGD revealed significantly higher DGR in 39 (78%) patients (p = 0.001). Pre- and post-operative changes in lymphocytes and plasma cells degree, polymorph infiltration of neutrophil in the lamina propria, loss of specialized glands from either antrum or body, intestinal metaplasia of the foveolar or surface epithelium and density of *H. pylori* overlying epithelium rates and statistical significances were demonstrated in Table 2.

**Table 1:** Demographic and clinical characteristics of patients

		Variables	p value
Age (years)		43 (25–84)	
Gender, M/F		0.51 (17/33)	
EGD, mean time (days)	Preoperative	38 ± 8	
	Postoperative	56 ± 10	
Presence of DGR (n, %)	Preoperative	24 (48%)	0.001*
	Postoperative	39 (78%)	
Presence of *H. pylori* infection (n, %)	Preoperative	14 (28%)	0.388*
	Postoperative	18 (36%)	
Sydney Score, mean value	Preoperative	2.38 ± 2.21	0.001**
	Postoperative	3.46 ± 3.05	

* Mcnemar’s test; ** Wilcoxon signed-rank test

**Table 2:** Pre- and post-operative endoscopic and histological inflammation scores

		Mild		Moderate		Severe		Total		p value
		n	%	n	%	n	%	n	%	
Inflammation	Preoperative	15	(30%)	3	(6%)	1	(2%)	19	(38%)	0.037
	Postoperative	15	(30%)	6	(12%)	3	(6%)	24	(48%)	
Activity	Preoperative	12	(24%)	6	(12%)	1	(2%)	19	(38%)	0.005
	Postoperative	22	(44%)	8	(16%)	2	(4%)	32	(64%)	
Atrophy	Preoperative	14	(28%)	3	(6%)	–	(–%)	17	(34%)	0.001
	Postoperative	13	(26%)	9	(18%)	2	(4%)	24	(48%)	
Intestinal metaplasia	Preoperative	20	(40%)	3	(6%)	–	(-%)	23	(46%)	0.157
	Postoperative	15	(30%)	6	(12%)	1	(2%)	22	(44%)	
*H. pylori* density	Preoperative	8	(16%)	4	(8%)	2	(4%)	14	(28%)	0.248
	Postoperative	12	(24%)	4	(8%)	2	(4%)	18	(36%)	
Sydney score	Preoperative	41	82(%)	8	(16%)	1	(2%)	–	–	0.109
	Postoperative	38	(76%)	10	(20%)	2	(4%)	–	–	

* Wilcoxon Signed-rank test

Postoperative pathological samples revealed that degree of inflammation, neutrophil activation, and glandular atrophy was significantly higher than the preoperative results. Chronic inflammation degree significantly showed an increase in 15 (30%) patients, decrease in 7 (14%) patients and remained as the same in 8 (16%) patients after laparoscopic cholecystectomy procedure (p = 0.007). Neutrophil activation degree was significantly higher in 21 (42%) patients, lower in 5 (10%) patients after the surgery (p = 0.005). Postoperative glandular atrophy degree was also higher in 13 (26%) patients and equal in 37 (74%) patients (p = 0.001). Pre- and postoperative degree of intestinal metaplasia and *H. pylori* density did not any show significant difference (p = 0.157, p = 0.248, respectively). Postoperative *H. pylori* infection was reported in 18 (36%) patients. Pre- and post-operative degree of *H. pylori* density was assessed and graded in moderate and severe in 4% each, and mild in 16, 24% of a total of 28 and 36% cases with atrophic changes, respectively. Postoperative *H. pylori* infection presented positive correlation with postoperative gastric mucosal changes including chronic inflammation (r = 0.41, p = 0.003), neuthrophil activation (r = 0.49, p = 0.001), glandular atrophy (r = 0.30, p = 0.03), intestinal metaplasia (r = 0.48, p = 0.001). However, postoperative *H. pylori* infection did not show significant correlation with postoperative DGR (r = 0.08, p = 0.55). In regard with these findings, postoperative Updated Sydney score mean value was significantly increased to 3.46 ± 3.05 (range 0–12, p < 0.000). However pre- and post-operative severity of gastric inflammation according to Updated Sydney score classification was not significant in between mild, moderate and severe grades of disease (p = 0.109).

## Discussion

DGR is physiological in humans (Ritchie 1984). Thus, reflux gastritis is found to be associated with the amount of bile refluxate. Antral biopsies revealed decreased and even no gastritis in patients with post-cholecystectomy DGR (Farsakh et al. 1995). In contrast, Wilson et al. have concluded that excessive DGR after cholecystectomy is found to be associated with persistent dyspeptic symptoms which correlated with high levels of bile refluxate and chronic gastritis on endoscopy (Wilson et al. 1995). Larusso et al. have also mentioned in a long term prospective study with ten patients that fasting bile reflux of total bile acids and DGR related gastritis increase progressively following the cholecystectomy procedure during the postoperative period (Lorusso et al. 1992). However, early onset of post-cholecystectomy DGR has presented comparable results with long-term reflux gastritis. Elhak et al. have found an increase in inactive form of chronic superficial gastritis and reflux gastritis on postoperative first year (Gad Elhak et al. 2004). Aprea et al. have evaluated elderly cholecystectomized patients with control endoscopy on postoperative 6 months and have concluded cholecystectomy is a significant risk factor for biliary gastritis (Aprea et al. 2012).

Post-cholecystectomy DGR related early chronic atrophic changes of gastric mucosa have not been clearly identified. DGR and mechanisms of gastric mucosal injury was shown in several in vitro and in vivo animal studies (Eastwood 1975; Stein et al. 1999; Nogi et al. 2001). The destructive effect of duodenal content over the gastric mucosa is defined as reactive gastritis (Sobala et al. 1993). Foveolar hyperplasia, oedema and congestion of lamina propria, acute and chronic inflammatory cells are the distinctive histopathological findings linked to reflux gastritis. Dixon et al. concluded that, prolonged exposure to the bilious duodenal content worsens mucosal injury and initiates endoscopic and histopathological sequence of gastric mucosal changes as atrophy, intestinal metaplasia and dysplasia (Dixon et al. 1986). *H. pylori* has been strongly accepted as a pathogen and a precursor of gastric mucosal transformation in each of these stages. Although bile reflux into the stomach is a potential inducer of intestinal metaplasia (Houghton et al. 1986), synergistic effect of the underlying *H. pylori* gastritis is stated as an essential factor in development of gastric mucosal changes (Sobala et al. 1991, 1993). The role of *H. pylori* infection in gallstones has been clearly demonstrated in several studies (Takahashi et al. 2014; Zhang et al. 2015). However, there is an ongoing debate about *H. pylori* colonization and its effects over the gastric mucosa in patients with DGR following the cholecystectomy procedure. Many studies mentioned that post-cholecystectomy DGR initiates gastric mucosal injury and reduces *H. pylori* colonization (Farsakh et al. 1995; Gad Elhak et al. 2004; Sobala et al. 1993; Atak et al. 2012). In contrast to these studies there are such reports demonstrated that patients with DGR after cholecystectomy have been presented with higher *H. pylori* colonization rates (Zullo et al. 1998).

In present study, early postoperative endoscopy revealed significantly higher rates of DGR and DGR related superficial chronic gastritis. Postoperative histological interpretations displayed increased *H. pylori* density, neutrophil activity, mucosal inflammation and atrophy degree. Although the Updated Sydney scores did not show significant difference between mild, moderate and severe groups, increased postoperative mean values were correlated with clinical outcomes. In this group of patients without *H. pylori* eradication therapy, postoperative pathological DGR and presence of *H. pylori* infection were found to be independent risk factors in development of early atrophic changes. These data suggested that early gastric mucosal atrophy following cholecystectomy procedure is possibly related to not only early onset of pathological DGR and excessive amount of bile refluxate but also presence of active *H. pylori* gastritis. Underlying *H. pylori* infection and DGR were thought to be the accompanying factors which potentiate the efficacy of each other’s detrimental effects over the gastric mucosa.

Limitations to our study depend on the challenges in determining the postoperative development of gastritis process and exact timing of histological sampling. Most of the adult population requiring cholecystectomy procedure have concomitant gastrointestinal disorders including *H. pylori* gastritis and DGR. Therefore, it is difficult to identify cholecystectomy as a sole risk factor for early mucosal atrophy. As indicated in our study, defining the exclusion criteria and evaluating the cumulative effects of DGR, underlying *H. pylori* gastritis and cholecystectomy procedure over the gastric mucosa should be more effective to specify the significant postoperative outcomes. Although gastric mucosal injury advances stepwise in a long time interval, postoperative early sampling of gastric mucosa should also reveal additional information about progression of preoperative existing lesions.

In conclusion, our study demonstrated cholecystectomy as a cause of severe DGR. Early onset of pathological DGR was also strongly associated with early gastric mucosal changes. Underlying *H. pylori* infection may be the most effective factor in development of early mucosal atrophy. These independent factors influence the predisposition to DGR related chronic atrophic changes. In regard to these findings, preoperative endoscopy should be performed to reduce the additive adverse effects of *H. pylori* gastritis and DGR over the gastric mucosa by managing the appropriate medical therapy during the preoperative course.
